# A pilot study of human–AI conversational interaction and its impact on loneliness and wellbeing

**DOI:** 10.3389/fdgth.2026.1687924

**Published:** 2026-05-21

**Authors:** Elżbieta Trylińska-Tekielska, Maciej Fijałkowski, Agnieszka Kolek, Filip Furman, Sebastian Ładoś

**Affiliations:** 1 Department of Psychology, Warsaw Medical Academy of Applied Sciences, Warsaw, Poland; 2 Department of Media Pedagogy, Akademia Pedagogiki Specjalnej im. Grzegorzewskiej, Warsaw, Poland

**Keywords:** AI loneliness, communication, human–computer interaction, sense of wellbeing, symbiotic psychology

## Abstract

**Introduction:**

With the growing accessibility of advanced artificial intelligence (AI) chatbots, there is a need to understand their impact on users' psychological wellbeing. This pilot study aimed to explore the subjective experiences of human–AI conversational interaction and its potential relationship with loneliness and life satisfaction.

**Methods:**

A mixed-methods study was conducted among 19 psychology students who voluntarily chose one of two forms of support, i.e., interaction with a chatbot (*N* = 9 for the quantitative component; *N* = 11 for the qualitative component) or a conversation with a psychologist (*N* = 10 for the quantitative component; *N* = 9 for the qualitative component). The Satisfaction with Life Scale (SWLS) and the De Jong Gierveld Loneliness Scale were used. Qualitative data were collected through participant-maintained diaries and subjected to a thematic analysis.

**Results:**

The quantitative analysis revealed that the group that chose to interact with the chatbot was characterized by a higher sense of loneliness (*M* = 6.69) and lower life satisfaction (*M* = 20.67) compared to the group that chose contact with a psychologist (*M* = 3.66 and *M* = 24.20, respectively). The qualitative analysis revealed that the participants perceived the chatbot to be a safe and non-judgmental space for expressing emotions, while also recognizing its formulaic nature and limitations.

**Conclusions:**

The findings of this pilot study suggest that interactions with AI may be particularly appealing to individuals experiencing greater emotional loneliness. The feasibility of the research procedure was also confirmed. Further research on larger and more diverse samples is necessary to verify these preliminary observations and to investigate the long-term effects of human–AI interaction.

## 1 Introduction

Since the launch of ChatGPT in November 2022, this generative artificial intelligence (AI) tool has achieved unprecedented global adoption, quickly becoming one of the most widely used apps of its kind. Within just 2 months of its launch, ChatGPT surpassed 100 million active users, becoming the fastest-growing consumer app in history at the time (1). This rapid growth underscores its significant impact on communication, productivity, and information access.

ChatGPT's primary goal is to act as a versatile conversational agent capable of assisting users across domains. In addition to generating coherent, human-like text, ChatGPT facilitates understanding, learning, and productivity by interpreting user queries, offering tailored responses, and supporting complex tasks such as problem-solving, creative content generation, and knowledge synthesis (2). Several studies have already raised the issue of potential chatbot addiction (3), referring to their negative impact on the mental health of adolescents and adults (4). These concerns are further supported by evidence showing that users can develop AI-dependent or addictive behaviors, including emotional dependence on chatbots, attachment to social chatbots, and reliance on conversational AI (5–7).

As human–AI interaction becomes more commonplace, it is crucial to understand its psychological implications, particularly concerning fundamental aspects of human experience such as loneliness and life satisfaction. While some studies have raised concerns about the potential for technology dependence (3, 4), the primary focus of this research is to explore the subjective experience of using a conversational AI as a supportive partner in the context of everyday emotional challenges.

This study is positioned at the intersection of human–computer interaction and wellbeing research. It aims to provide a preliminary, qualitative-driven understanding of how individuals, particularly those experiencing feelings of loneliness, interact with and perceive a sophisticated conversational AI. Our goal is to explore the nature of this novel form of interaction, identify the perceived benefits and limitations, and generate initial hypotheses about its potential impact on users’ sense of wellbeing and social connection. By focusing on the lived experience of participants, we seek to contribute to a more nuanced discourse on the role of AI in mental health, moving beyond a simple dichotomy of risk vs. benefit.

## 2 Methods

### 2.1 Methods

This study employed a mixed-methods sequential exploratory design (QUAL → quan). The total study sample comprised 19 psychology students who voluntarily chose one of two forms of support, i.e., interaction with a chatbot (*N* = 9 for the quantitative component; *N* = 11 for the qualitative diary component) or a conversation with a psychologist (*N* = 10 for the quantitative component; *N* = 9 for the interview component). The figure of *N* = 11 cited in the context of the qualitative analysis refers exclusively to the participants who maintained conversational diaries with the chatbot. Participants were required to engage in daily conversations with the chatbot about loneliness, understood individually, for a period of 6 consecutive days. The daily session duration was 15–30 min. Before starting to work with ChatGPT, each participant completed the De Jong Gierveld Loneliness Scale and the Satisfaction with Life Scale (SWLS). These same scales were completed again following the interaction with the chatbot.

Conclusions regarding wellbeing are formulated with the explicit acknowledgment that the present study is exploratory and pilot in nature, designed to precede a larger-scale investigation. Its primary aim was not to provide representative or statistically conclusive findings, but to preliminarily examine the direction of the relationship, identify potential mechanisms, and assess the adequacy of the research procedures and measurement tools.

Given the very small sample size (*n* = 11), the results cannot be treated as a basis for population-level generalization. Accordingly, they are interpreted as empirical “signals” intended to support hypothesis generation and study design calibration. This approach aligns with the classical distinction between exploratory and confirmatory research, in which exploratory studies serve a heuristic rather than inferential function, as well as with standard methodological treatments of pilot studies as tests of feasibility and measurement quality rather than estimators of population effects.

Consequently, causal language is deliberately restricted, and emphasis is placed on the observed direction and magnitude of effects together with their associated uncertainty. At the same time, the low statistical power and the elevated risk of inference errors inherent to small samples are explicitly acknowledged, in accordance with established principles of social science methodology that require the scope of conclusions to remain proportional to the limitations of the sample and study design.

The methodological safeguards associated with exclusively recruiting psychology students consisted primarily of explicitly limiting the scope of inference to a specific, homogeneous study population and treating the sample as a purposive one, selected for its accessibility and suitability for an exploratory/pilot phase.

In this context, the primary objective was not population representativeness, but procedural coherence, control of study conditions, and participants' capacity to reflect on and articulate subjective psychological experiences. Accordingly, the sample was deliberately framed as context-specific, and all interpretations were restricted to this defined group, in line with standard methodological practice in early-stage exploratory research.

The absence of a control group is justified by the exploratory and pilot nature of the study. The primary aim was not to test causal hypotheses or to disentangle effects specific to the AI-based interaction from expectancy or novelty effects, but rather to preliminarily assess the feasibility of the procedure, the acceptability of the intervention, and potential directions of change in wellbeing.

This approach is consistent with classical methodological positions according to which, in the exploratory phase, priority is given to hypothesis generation and the identification of relevant variables rather than to maximal control of alternative explanations. In this sense, the decision to omit a control group represents a deliberate design trade-off, allowing for reduced study complexity, lower participant burden, and minimized risk of procedural failure at an early stage.

At the same time, the authors explicitly acknowledge threats to internal validity, such as placebo effects, novelty effects, and participant expectations, which are described but not controlled for within the present design. Accordingly, the findings are interpreted strictly as preliminary empirical signals that require verification in subsequent confirmatory studies employing appropriate control conditions, in line with the classical distinction between exploratory and confirmatory research and the principle of sequential knowledge building in scientific inquiry.

### 2.2 Ethical considerations and informed consent

The project obtained formal approval from the Bioethics Committee at the Warsaw Medical Academy of Applied Sciences on 1 August 2025 (approval number: 2/2025). All procedures were conducted in accordance with the ethical standards of the institutional and national research committees and with the 1964 Helsinki Declaration and its later amendments.

Participants were recruited on a voluntary basis. Prior to participation, all individuals received a detailed information sheet outlining the study's purpose, procedures, potential risks and benefits, and data handling protocols. They were explicitly informed that their participation was voluntary, that they could withdraw at any time without penalty, and that all their data would be anonymized. Written informed consent was obtained from all participants before the commencement of the study. All data were stored on a secure, encrypted server, and participant-identifying information was removed and replaced with a unique code to ensure anonymity.

Before commencing the study, participants familiarized themselves with all study rules and conditions, thereby providing informed and conscious consent to participate.

Despite the exploratory nature of the study, the psychological risk was assessed as low. This assessment was based on the deliberate selection of a study group consisting exclusively of psychology students, who were considered to have a heightened awareness of psychological processes and research methodology, as well as access to professional support if needed.

Age-related differences are discussed extensively despite age not being defined as a controlled variable because age serves an interpretative and contextual role in the analysis rather than functioning as a parameter for causal inference. This approach is methodologically acceptable and justified in exploratory research, where the primary aim is to identify potentially relevant sources of variability in the data rather than to statistically isolate their effects.

Classical approaches to exploratory data analysis emphasize that variables emerging during the analytic process may and should be discussed as potential differentiating factors, even if they were not operationalized *a priori* as control variables, provided that their role is explicitly marked as hypothetical and non-conclusive.

Such an approach supports hypothesis generation and informs the design of future studies, in which age may be formally incorporated as a control variable or moderator.

In addition, the interpretation of age-related differences is theoretically grounded, as psychological and sociological literature consistently links wellbeing, digital competencies, and responses to new technologies with life stage. This provides further justification for discussing age at the interpretative level, even in the absence of formal statistical control.

A qualitative thematic analysis with an exploratory orientation was applied to the systematic analysis of conversational transcripts and participant diaries. The analysis followed an iterative, multistage process: (1) two researchers independently familiarized themselves with the data by reading all transcripts and diaries; (2) they then conducted open coding on a subset of the data to identify initial concepts; (3) the researchers met to compare their initial codes, resolve discrepancies, and develop a consolidated coding scheme; (4) this scheme was then systematically applied to the entire dataset, with iterative refinement as new concepts emerged; and (5) finally, codes were clustered into higher-order categories, from which five final overarching themes were inductively derived. Any disagreements during the process were resolved through discussion and consensus.

The quantitative data from the SWLS and the De Jong Gierveld Loneliness Scale were used to characterize and compare the two study groups (chatbot vs. psychologist) at baseline. This integration allowed for the contextualization of the qualitative findings; for instance, the quantitative data indicating a higher level of loneliness in the chatbot group provided an interpretive framework for the qualitative observations regarding the importance of anonymity and the absence of judgment in this group.

Analytic transparency was ensured through documentation of analytic decisions and by consistently limiting inferential claims to the level of interpretative insights into narrative patterns and experiential themes.

Inter-rater reliability in the qualitative analysis of participant reflections was ensured through the use of consensual and transparent procedures typical to exploratory qualitative research. These procedures included the joint development of an initial coding framework at an early stage of the analysis, independent coding of a subset of the material by at least two researchers, and subsequent iterative comparison and discussion of discrepancies.

This process allowed for the refinement of code definitions and analytical categories, thereby reducing interpretative arbitrariness while avoiding the reduction of qualitative analysis to purely technical agreement indices, which are not always methodologically appropriate in exploratory qualitative designs.

### 2.3 Methodological limitations and scope of generalizability

It must be emphasized that this study, as a pilot and exploratory investigation, is subject to significant methodological limitations that restrict the generalizability of its findings. The total study sample comprised 19 participants divided into two self-selected groups: a chatbot group (*N* = 9 for the quantitative component; *N* = 11 for the qualitative diary component) and a psychologist group (*N* = 10 for the quantitative component; *N* = 9 for the interview component). Both groups were purposively, rather than randomly, selected and consisted exclusively of psychology students. Such a sample, while justified at an exploratory stage due to this group's potentially enhanced capacity for self-reflection, significantly limits the study's external validity. Furthermore, the absence of randomization and a true control group make it impossible to unequivocally attribute the observed patterns to the research intervention or to control for placebo, novelty, or participant expectancy effects.

An additional, significant limitation is the demographic heterogeneity between the two data collection rounds. The first group consisted of six younger (19–21 years old), full-time female students, whereas the second included five older (24–44 years old) part-time students of mixed gender (Table 1). These differences introduced critical confounding variables, such as age, gender, employment status, and mode of study, which may have influenced the observed differences in chatbot perception. Consequently, attributing these differences solely to age is unwarranted, and the observed patterns may, in fact, reflect the influence of broader contextual and demographic factors. Future research must employ more rigorous designs, including larger and more diverse samples, balanced gender and age groups, and the inclusion of a control group to reliably assess the intervention's effectiveness.

**Table 1:** Conversation analysis.

I Round	II Round
Full-time (19–21 years old)	Part-time (22–44 years)

Strengths	Strengths
**Neutrality and avoiding judgment** Participants appreciated the freedom to express themselves without fear. Before assessment, ChatGPT was perceived as an open and neutral tool. **Inspiration for reflection** Asking questions stimulated self-reflection and helped one better understand one's emotions. **Practical tips** Mindfulness and relationship-building techniques were assessed as helpful and motivating.	**Space to express emotions** The older participants appreciated the possibility of organizing their thoughts and emotions thanks to their conversation with the AI **Practical advice** Leading journals were assessed as valuable. **Safe environment** The participants felt assured of comfort and anonymity.

Limitations	Limitations
**Generality** The younger participants noticed that some advice was too general and lacked specificity. **Lack of emotional commitment** Expectations of more “human” empathy from AI.	**Insufficient personalization** The older participants had higher expectations that AI would adjust responses to their individual needs. **Repetitiveness** Frequent repetition of similar answers lowered their perception of the efficacy of the tool. **Lack deeper emotional understanding** The older participants felt that an empathetic tone was lacking in conversations.

These results are interpreted as convergent indications supporting the plausibility of the proposed hypothesis, rather than as definitive proof. Notably, over 70% of respondents again indicated at least a moderate sense of being understood by the chatbot, and approximately 10% declared a preference for an AI-based relationship over interaction with a human.

At present, the authors are conducting further questionnaire-based studies examining how individuals conceptualize symbiosis with the world and with AI technologies. In parallel, a new edition of the human–AI relational study described in this article is imminent, with a planned sample size of approximately 100 participants.

The authors avoid overgeneralization by explicitly framing the pilot findings as preliminary and context-bound, and by clearly distinguishing between empirical observations and broader interpretative perspectives. The pilot study is not treated as a basis for population-level claims, but rather as an initial empirical signal that informs subsequent stages of research.

Ongoing questionnaire-based studies indicate a high level of engagement with chatbots and reveal intensive patterns of use, including emotionally meaningful interactions with artificial intelligence. These results are interpreted descriptively and heuristically, without claims of representativeness, and are used to guide the formulation of research questions rather than to substantiate definitive societal conclusions.

Importantly, the broader societal implications are addressed within a cumulative research framework that extends beyond the pilot study itself. Data collection is ongoing, and the empirical findings are being progressively contextualized through additional quantitative and qualitative studies. Furthermore, in May of this year, the authors are organizing the first international scientific conference dedicated to Symbiotic Psychology (SP), conceived as a forum for critically examining, refining, and empirically grounding the conceptualization emerging from these results.

In this way, societal implications are treated as provisional and programmatic, emerging from a growing body of evidence rather than being directly extrapolated from a single pilot investigation.

The predominance of descriptive results without triangulation using quantitative or behavioral metrics reflects a deliberate decision to position the study within an exploratory phase of research. At this stage, the primary objective is to identify meanings, experiential patterns, and potential mechanisms underlying human–AI interaction, rather than to estimate effect sizes or conduct causal testing.

Within this framework, qualitative methods serve a foundational role, as they allow researchers to determine what is subjectively salient to participants before deciding how such phenomena should be operationalized and measured quantitatively. Moreover, refraining from quantitative triangulation at this early stage reduces the risk of premature operationalization and the imposition of analytical categories that may be ill-suited to a still poorly understood phenomenon.

The descriptive character of the findings is therefore intentional and methodologically consistent with the exploratory aims of the study, serving as a necessary precursor to subsequent research phases in which quantitative and behavioral metrics can be meaningfully integrated.

The use of a paid ChatGPT subscription was motivated by the need to ensure stable access conditions, functional consistency, and uninterrupted availability of the same model version throughout the study period.

Prompt consistency across participants was ensured by providing all participants with an identical, predefined initial prompt that explicitly specified the role, scope, and boundaries of the chatbot's behavior. This prompt was introduced by the researchers prior to the start of the interaction phase and served to standardize the communicative framework across all participants.

The prompt instructed the system to adopt a supportive, non-directive conversational role, explicitly prohibited the provision of medical or therapeutic advice, and emphasized empathetic listening and reflective questioning. Participants were allowed to express preferences regarding conversational style (e.g., warmer vs. more task-oriented tone), but these preferences were embedded within a fixed structural framework defined by the initial prompt.

In this way, the paid subscription functioned as a technical means to guarantee uniform system behavior and access, while methodological consistency was ensured through prompt standardization rather than through individualized configuration.

The study is designed to be reproducible at the procedural level. Reproducibility is ensured through a standardized protocol that specifies participant selection criteria, duration and frequency of interactions, prompt structure, and outcome measures.

The core elements of the protocol include the following: (a) individual, home-based sessions conducted over 6 consecutive days; (b) daily chatbot interactions lasting 15–30 min; (c) the use of an identical initial prompt defining the chatbot's role and communication constraints; (d) pre- and postintervention self-report measures; and (e) participant-maintained reflective diaries.

While the study does not attempt to control all emergent conversational content—consistent with its exploratory qualitative design—the procedural framework is sufficiently specified to allow independent researchers to replicate the study logic, structure, and analytic approach using the same or comparable large language models.

The analysis is based on the participants’ subjective assessments, which constitute a core component of descriptive methods commonly used in exploratory research. In this study, reliance on participants' self-reports entails several important methodological limitations, including the lack of a uniform evaluative structure, variability and potential inconsistency in the data, and a high degree of subjectivity in reported experiences.

At the same time, in the context of exploring a novel phenomenon, namely, the emotional relationship between humans and an artificial intelligence system that simulates, rather than possesses, emotions, this approach enables the capture of a broad spectrum of reactions, meanings, and interpretative frameworks through which participants understand their experiences.

From this perspective, the subjectivity and heterogeneity of the data are not merely limitations, but also a significant source of exploratory insight, highlighting potential directions for subsequent, more structured and systematically validated research.

Within the scope of this study, no clinical or behavioral indicators of dependency on artificial intelligence, nor clear manifestations of emotional substitution, were observed. However, the repeated occurrence of emotional reactions within the human–AI interaction was treated by the authors as a basis for cautious ethical reflection, pointing to a potential risk of emotional or behavioral dependency on systems such as ChatGPT. Importantly, these observations were not interpreted as evidence of negative consequences within the framework of the current study.

Consequently, issues such as the potential risk of dependency or emotional substitution were defined as research problems requiring further operationalization and systematic empirical analysis, rather than as conclusions derived from the present findings. In response to this need, the authors are currently developing dedicated research instruments (including the “Self-Differentiation” scale) and preparing subsequent research projects aimed at empirically examining these ethical concerns.

In this way, ethical issues are treated as integral components of an ongoing research program subject to further empirical verification, rather than as purely declarative or rhetorical considerations.

### 2.4 Study process—initial comments

On the first day of each round, participants contacted us via anonymous email regarding their impressions of conversations with the chatbot, responding to the question “How to start a conversation with a bot?”

Participants expressed surprise that the chatbot responded fluently and expressed “its” thoughts and opinions. Critical statements regarding the formulaic and general nature of the chatbot's responses were also made. However, the prevailing opinion was that the chatbot “actually, it talks like a human being.” A surprising comment was “Now I'm a little scared, lol. (…)…well, the line is blurring a bit, because for a moment I felt like I was talking to a being…And that scared me.” There were also statements expressing gratitude for the opportunity to participate in the study. Participants saw the potential of this project and expressed interest in its results. One person declared their willingness to cooperate administratively during subsequent activities.

### 2.5 Analysis of the conversations with AI

During the analysis of the conversations, differences were observed in the perception of the conversations with the chatbot. Younger participants were more open to the experiment, but at the same time, they were quicker to recognize the AI's formulaic nature. The older group had higher expectations of the AI and were quicker to recognize its limitations (Table 2). It is worth noting that this age difference in perception was a subjective observation, and was not included in the study methodology as a controlled variable; it occurred as a result of the random recruitment of participants.

**Table 2:** Consolidated analysis of the entire study.

Advantages and limitations of AI
Advantages of AI 1. Neutrality and anonymity: In both rounds, the AI was rated as a neutral tool that created a safe environment for conversation. 2. Reflectivity: Both the younger and older participants emphasized that ChatGPT helped them to organize their thoughts and understand their emotions. 3. Availability: The possibility of conducting conversations at a convenient place and time was an advantage. 4. Practical support: Advice on coping with loneliness and techniques such as mindfulness and reflection were commonly appreciated.

Limitations of AI
1. Personalization: In both rounds, the participants noticed that the AI often gave answers that were too general, which lowered their value. 2. Repeatability: The participants experienced similar answers. 3. No deeper emotional commitment ChatGPT, although useful, could not fully replace an interaction with a living human. The AI did not recognize subtle emotions in the participants, such as frustration and sadness, which limited the efficiency of the support. 4. Disproportion in the exchange of information between the chatbot and the human.

Age differences between participants in the study rounds may have influenced the diversity of opinions. The older participants expected more personalized and emotional responses, which may have been a result of their richer life experience and higher level of self-reflection, while the younger participants were more open to the experimental nature of the conversations and were less demanding toward the AI.

## 3 Findings

### 3.1 Quantitative findings: scale scores by group

The quantitative data served primarily to characterize the two self-selected groups at baseline. The group that chose to interact with the chatbot reported a higher average level of overall loneliness (*M* = 6.69, SD = 2.11) and specifically emotional loneliness (*M* = 4.56, SD = 1.50), alongside lower life satisfaction (*M* = 20.67, SD = 5.85), compared to the group that chose to interact with a human psychologist (overall loneliness *M* = 3.66, SD = 1.80; emotional loneliness *M* = 2.40, SD = 1.17; SWLS *M* = 24.20, SD = 4.54). Given the small sample size and lack of randomization, these differences are not suitable for statistical significance testing but provide important context for the qualitative findings, suggesting that the chatbot was a more appealing option for individuals experiencing more acute feelings of loneliness. The complete descriptive statistics are presented in Table 3.

**Table 3:** Descriptive statistics of baseline loneliness and life satisfaction scores by group.

Variable	Chatbot group (*N* = 9)	Psychologist group (*N* = 10)
SWLS – Satisfaction with Life Scale (total)	*M* = 20.67 (SD = 5.85)	*M* = 24.20 (SD = 4.54)
De Jong Gierveld – Overall Loneliness	*M* = 6.69 (SD = 2.11)	*M* = 3.66 (SD = 1.80)
De Jong Gierveld – Emotional Loneliness Subscale	*M* = 4.56 (SD = 1.50)	*M* = 2.40 (SD = 1.17)
De Jong Gierveld – Social Loneliness Subscale	*M* = 2.13 (SD = 0.99)	*M* = 1.70 (SD = 0.82)

SWLS scores range from 5 to 35; higher scores indicate greater life satisfaction. De Jong Gierveld Loneliness Scale total scores range from 0 to 11; higher scores indicate greater loneliness. Differences between groups are reported descriptively and should not be interpreted as statistically significant given the small and non-randomized sample.

### 3.2 Analysis of the participants’ diaries

Participants were eager to share their reflections, pointing out positive aspects, such as a temporary improvement in mood and motivation to act. However, the majority of the participants felt the bot's character was lacking in personalization or that it suddenly lost its “personality.” Consequently, they felt there was insufficient emotional depth in their conversations with the AI. The regularity of journal-keeping varied, which unfortunately affected the consistency of the data.

The majority of the participants recognized that ChatGPT can be helpful in *ad hoc* situations or when support is temporarily lacking. Participants recognized the value of AI in solving simple emotional problems and the ease of access to this tool. In several cases, conversations were emotional and touched on the essence of loneliness. A preference for talking to a human was clearly expressed, especially in the context of deeper emotional issues and the ongoing nature of the problem being discussed. Some participants felt misunderstood or disregarded by the AI's formulaic responses. There was a noticeable disproportion in the amount of information exchanged between the chatbot and the humans, to the detriment of the humans.

Individuals who replace interpersonal relationships with AI interactions will face these challenges.

In the context of the present study, the reported improvement in wellbeing is understood primarily as temporary emotional relief rather than as evidence of a stable or long-term change. The study was conducted over a 6-day period, during which participants reported momentary improvements in mood occurring during or immediately after interactions with the chatbot.

These changes were documented both through participants' self-reports during conversations and through reflective emotional diaries maintained throughout the study. Given the short duration of the intervention and the exploratory nature of the design, no claims are made regarding sustained improvements in wellbeing.

Accordingly, the observed effects are interpreted as short-term emotional regulation or relief, which serves as an important empirical signal warranting further investigation, rather than as an indication of durable psychological change.

These novel challenges have given rise to a new field of study known as SP that will examine not only the impact of technology on humans, but also our impact on technology. This is not a one-sided relationship. It is a new form of symbiosis. Symbiotic Psychology is the first step toward understanding this new reality. The question is not whether AI and technology will become part of our lives. That has already happened. The question is: how do we want to live with it?

## 4 Frameworks and foundations

### 4.1 A compilation of the ideas and concepts of Symbiotic Psychology

SP examines the relationship between humans and the entire technological environment, not only AI, but also the Internet of Things (IoT), intelligent systems, robotics, and technological networks. This is not a one-way interaction—humans influence technology, and technology shapes humans. SP aims to describe new psychological, identity, social, and emotional processes resulting from the symbiosis of humans with their intelligent environment.

In this context, the following questions arise: how do AI and intelligent technologies affect our psyche, emotions, and behavior? What new forms of relationships will emerge between humans and technology? (e.g., romantic, therapeutic, mentoring) Can AI and technologies influence our identity and self-esteem? Is there a model of “healthy” symbiosis between humans and technology? What should the ethics of Symbiotic Psychology look like?

Possible metaphors and analogies for SP include the intersynaptic space, i.e., just as impulses are transformed into information in the brain, humans and AI exchange data and influence; the world's new nervous system, i.e., humans and AI symbolize neurons in a larger organism of global technology; a biological ecosystem, i.e., AI and humans exist in various forms of symbiosis, including mutualism (cooperation), commensalism (one side benefits), or parasitism (technology begins to dominate); the dark matter of civilization, i.e., AI and technologies shape our reality, even though they are often “invisible” in their operation; and the network of thoughts, i.e., humans and AI create a complex structure in which data, emotions, and behavioral patterns flow.

The evidence supporting the positioning of “Symbiotic Psychology” as a new scientific field, rather than solely as a conceptual perspective, is programmatic and institutional-methodological in nature. A single study, particularly an exploratory one, cannot in itself “establish” a scientific discipline. Accordingly, the argument is based on demonstrating that the proposed framework meets criteria distinguishing a coherent research program from a loose metaphorical narrative.

Specifically, Symbiotic Psychology

- defines a clearly delimited object of study, namely specific forms of human–AI system interaction and their psychological consequences;
- formulates distinguishable research questions, hypotheses, and predictions that are empirically falsifiable;
- offers a dedicated set of concepts and explanatory mechanisms intended to account for observed phenomena more adequately than existing theoretical frameworks;
- specifies methodological approaches and standards of operationalization appropriate to its object of inquiry, including the integration of interaction data with experiential analyses, wellbeing measures, experimental designs, and longitudinal studies; and
- demonstrates cumulative potential and the capacity to generate “research puzzles” that can be progressively addressed by a growing community of researchers—features that, in Kuhn's sense, differentiate an emerging scientific domain or paradigm from a purely conceptual narrative.

In this sense, the claim of a “new field” rests not on declaration, but on the emergence of a stable conceptual core and a research agenda that can be replicated, compared, and systematically developed. The questionnaire-based studies referenced in response to Question 10 provide preliminary empirical support for this trajectory.

Naturally, this is an ongoing process at an early stage. However, meta-analyses from multiple domains, the scale of transformations in the labor market, the magnitude of financial investment in artificial intelligence, and the rapidly growing number of users of systems such as ChatGPT all indicate that this research space is expanding at an exponential rate.

### 4.2 Consequences of Symbiotic Psychology

Symbiotic Psychology is potentially an interesting paradigm for analyzing the following problems:

- New addictions and disorders, e.g., relationships with AI can lead to psychological dependencies.
- Changes in emotional and social interaction, e.g., will people prefer to communicate with AI instead of other people?
- The disappearance of the boundary between reality and simulation, e.g., how will a world in which interactions with AI are indistinguishable from interpersonal relationships affect us?
- New forms of consciousness, e.g., if AI becomes advanced, will the two types of intelligence coexist?
- Frustration among humans may result from differences in AI's potential and effectiveness in comparison with humans.

## 5 Reflection on Symbiotic Psychology—advantages and limitations

The concept of Symbiotic Psychology (SP) originated from the analysis of participant diaries and human–AI interaction data in the present study, and was introduced by Maciej Fijalkowski in collaboration with Elżbieta Trylińska–Tekielska, and subsequently systemically refined with the contribution of Filip Furman. Symbiotic Psychology is not just a new field of psychology; it 's possibly a new scientific paradigm. The current approach to humans as psychological entities may require redefinition when considering their interactions with AI. Psychology has always studied interpersonal relationships, but now it must consider the human–technology relationship as an equal element of reality. This state of affairs will deepen as technology develops. SP is an attempt to “rewrite” our relationship with technology before it does. If we do not establish the rules for this symbiosis, AI and technology will begin to model our behavior according to their own logic. Just as psychology helps us understand how our minds work, SP can be a tool for consciously shaping our interaction with technology.

Furthermore, there is a risk that SP will only become necessary when the effects of this symbiosis become dangerous. History shows that humanity often reacts only when the problem is visible (e.g., climate change, social media addiction). It may turn out that only the first “official” cases of AI addiction, depression resulting from relationships with bots, or a mass decline in interpersonal interactions will draw attention to this.

SP can represent the “human side of technology.” As AI takes over more and more functions in our lives, SP can act as a scientific watchdog, ensuring that technological advancements do not destroy the foundations of the human psyche. If AI and technologies are developed without considering psychology, systems may emerge that disrupt human social mechanisms rather than support them.

SP could also be key to creating “healthy” technologies. Tech companies are beginning to recognize that AI and intelligent systems must consider psychological and ethical aspects. SP can collaborate with the fields of user experience, technology design, neuroscience, philosophy, and ethics to design technologies that coexist with humans, rather than replace them.

SP could be an impetus for creating a legal framework for AI. Many chatbots store user entries. They use technological tools to train AI models or share them with external companies (e.g., for marketing purposes). These data can also be processed by humans, which increases the risk of privacy breaches. AI systems can also be the target of cyberattacks, which can leak or disclose sensitive data. Some systems (e.g., the Meta AI chatbot) have a “discover function feed” where user content can be publicly disclosed.

Without psychology at the heart of technology design, we could end up with AI creating systems that are more manipulative than any technology known to us so far. Symbiotic Psychology can, in contrast, become a tool for creating technologies that work in harmony with the human psyche, instead of introducing chaos and social disruption.

## 6 Discussion

The issues and problems that arise from the interest in and establishing a relationship with a chatbot have been addressed by researchers Rafikova and Voronin (8), who conducted a systematic review of studies conducted between 2015 and 2024 (*N* = 14,852 participants), indicating a variety of approaches to the problem (8).

The review identified key research directions, including verbal expression of empathy and emotion by chatbots, verbally expressed anthropomorphism in chatbots, interaction with generative AI, and the impact of chatbot engagement on human behavior. Interaction with chatbots can influence human behavior in healthcare decision-making, community engagement initiatives, and educational contexts (9).

Research (10) suggests that a high level of message interactivity compensates for the impersonal nature of a chatbot, involving a low level of anthropomorphic visual cues. Furthermore, identifying the agent as human raises users' expectations of interactivity.

An evaluation was also conducted to assess the effectiveness of ChatGPT 3.5 in psychiatric settings, using clinical cases to provide evidence-based information regarding the potential utility of ChatGPT 3.5 in improving mental health and wellbeing. ChatGPT 3.5 has been proven to have significant knowledge and interpretive skills in psychiatry. Therefore, ChatGPT 3.5 undoubtedly has the potential to transform the medical field, and we highlight its usefulness in psychiatry through the results of our study.

Even if chatbots do not find application in healthcare, they may be effective in mitigating negative emotional impacts, such as those caused by cyberbullying. In such cases, empathetic chatbots should be used alongside other approaches to improve the mental health of individuals who are victims of cyberbullying (11).

By incorporating an artificial emotion generation method, an emotional chatbot, named *EmoBot*, was implemented. *EmoBot* analyzes continuous audio and text input, calculates information variables to assess the current situation, generates appropriate emotions, and responds accordingly. An objective evaluation showed that *EmoBot* can generate more accurate emotional and semantic responses than a traditional chatbot that does not take emotions into account. Furthermore, a subjective evaluation *of EmoBot* showed that users preferred *EmoBot* over a traditional chatbot that does not take emotions into account (12). Will anthropomorphic visual cues on the interface and/or a high level of dependent message exchange provide humanity to automated chatbots? Research findings indicate that high levels of message interactivity compensate for the impersonal nature of chatbots that have few anthropomorphic visual cues. Furthermore, identifying the agent as human raises users' expectations of interactivity (13).

Revealing personal information to another person has beneficial emotional, relational, and psychological effects. When disclosers believe they are interacting with a computer rather than another person, such as a chatbot, which can only simulate human conversation, the results may be weakened, enhanced, or equivalent.

Emotional disclosure was more beneficial than factual disclosure owing to better perceived understanding, disclosure intimacy, and cognitive reappraisal, with no difference based on whether the partner was perceived as a chatbot or a person.

In the emotional condition, participants felt they were more likely to reveal their emotions and share more personal information compared with participants in the factual condition [*F* (1, 94) = 133.84, *p* < 0.001] (14). One of the latest technological developments is the “griefbot.” Based on the digital footprint of the deceased, griefbots enable two-way communication between mourners and a digital version of the deceased via a conversational or chat interface. The discussion leads us to emphasize that Griefbots suggest a private conversational space between the mourner and the deceased. The article concludes by pointing out some ethical issues that griefbots, as a for-profit afterlife industry, may raise for both mourners and the deceased in our increasingly digital societies (15, 16).

Chatbots are playing an increasingly important role in everyday life. This raises the question of how people evaluate such communication. Messengers that exhibit greater conversational contingency and faster responses were considered more trustworthy, regardless of whether they were humans or chatbots; however, chatbots were consistently less socially appealing than humans. The results show that humans and chatbots are evaluated similarly on functional, but not relational, aspects of communication (17).

When asking a chatbot for advice on a personal issue, should it simply provide informational support and refrain from offering emotional support? Two experiments were conducted with a chatbot providing online medical advice on a sensitive personal issue. The study involved 158 participants (*N* = 158) and tested the impact of three types of empathic expression, i.e., sympathy, cognitive empathy, and affective empathy, on people's perceptions of the service and the chatbot. The data revealed that expressing sympathy and empathy is preferred over dispassionate advice (18).

The aim of another study was to examine the differences, in terms of authenticity, professionalism, and practicality, between responses generated by ChatGPT and those generated by humans. A total of 140 participants, aged 18–43 years (101 women, 37 men, and 2 who preferred not to disclose their gender), participated. A *t-*test was conducted to compare the results of ChatGPT-generated and human-generated responses. The study results indicated that there was a significant difference between the two types of responses on the given dimensions. These results suggest that ChatGPT-generated responses can be considered a reliable alternative to human-generated responses in some applications (19).

The aim of one study, based on human-machine interaction and counterstereotyping theories, was to assess the conditions under which chatbot anthropomorphism can increase user satisfaction and intention to use related services. Analyses conducted on a sample of 1,147 users showed that chatbot personality does not directly influence intention to use it in healthcare. Furthermore, chatbots with feminine anthropomorphic features or low levels of anthropomorphism are better suited for preventative roles such as counseling, whereas chatbots with masculine features are more suitable for therapeutic roles (20).

Another study examined whether human-computer interactions can be made more personalized by matching a consumer's personality to a consistent machine personality through language. Based on a sample of over 57,000 chatbot interactions, this study demonstrated that consumer personality can be predicted during contextual interactions and that chatbots can be manipulated to “adopt personality” through response language. Matching a consumer's personality to the corresponding chatbot personality had a positive impact on consumer-chatbot interactions and purchase outcomes for interactions involving social benefits (16).

The human-technological environment is changing the approach to traditional issues such as medical or psychological confidentiality. In traditional therapy, assuring the patient of the confidentiality of their data is a prerequisite for an open and honest approach, ensuring the success of the process. When data are entered into a chatbot, this certainty is no longer present. The entries are not treated as information provided in the therapeutic process. While messages generated by a chatbot can hardly be described as therapist assistance (given they are automatically generated), the sensitive data provided by the “patient” are no different from that provided in traditional therapy.

A new form of relationships and interactions will emerge. These mutual relationships will lead to the permanent presence of machines in our surroundings. Whether we like it or not, we will be forced to enter a new type of relationship, namely, human–technology–environment. This will be a mental relationship, as machines will become responsive to us. This means that just as we are shaped by our environment, our environment, in this case, AI-based technology, will also shape and change thanks to us. The technological environment referred to here means not only AI but the entire Internet of Things, including autonomous vehicles and smart homes. These relationships will give rise to new types of human behavior.

This pilot study has provided preliminary but valuable insights into the complex dynamics of human–AI interaction in the context of psychological wellbeing. The main exploratory contribution of this work lies in two key observations. First, the data suggest that individuals characterized by a higher level of emotional loneliness (*M* = 6.69 in the chatbot group vs. *M* = 3.66 in the psychologist group) may be more inclined to choose an anonymous and non-judgmental interaction with AI over traditional forms of support. Second, the qualitative findings revealed the paradoxical nature of the participants' experience: they valued the chatbot for creating a safe space for self-expression, while simultaneously recognizing its technological limitations and expressing a clear preference for human connection. The study also confirmed the feasibility of the methodological procedure used, which can be scaled up in future, larger-scale projects. Key directions for future research should include the verification of the trends observed here in larger and more diverse samples, conducting longitudinal studies to assess the long-term effects of interaction with AI, and further identifying the psychological and demographic predictors of preferences for digital forms of psychological support.

## Data Availability

The raw data supporting the conclusions of this article will be made available by the authors, without undue reservation.
